# Isolation and subtyping of avian influenza A virus from wild birds in Khartoum, Sudan

**DOI:** 10.4102/ojvr.v92i1.2228

**Published:** 2025-12-15

**Authors:** Wegdan H. Ali, Intisar K. Saeed, Shaza M. Mutwakil, Muzdalifa H. Alamin, Abdelgader A. Balla, Mona A.E. Ahmed, Abubaker A. Saeed, Rayan M. Asil, Omer A. Algezoli, Muaz M. Abdellatif, Yahia H. Ali

**Affiliations:** 1 Department of Virology, Central Veterinary Research Laboratory, Khartoum, Sudan; 2 Department of Biological Sciences, College of Science, Northern Border University, Arar, Saudi Arabia; 3 Wild life Research, Wild life Research Center, Khartoum, Sudan

**Keywords:** avian influenza A, subtype H5, isolation, wild birds, Sudan

## Abstract

Avian influenza (AI) is a significant disease affecting chickens and other avian species. Wild birds are thought to contribute to the virus transmission. The present study intends to explore the existence of AI type A virus in wild birds at the Six April Zoo, Khartoum State, Sudan. A total of 42 cloacal and tracheal swabs were collected from clinically healthy individuals belonging to five different wild bird species. The selected wild bird species were Common crane *Grus grus*, Sudan crowned crane *Balearica pavonina*, Helmeted guinea fowl *Numida meleagris*, Duck sp. *Anatidae* and Chestnut-billed sand grouse *Pterocles exustus*. Swabs were examined for AI virus antigen using the agar gel immunodiffusion (AGID) test, and all tested swabs produced positive results. The swab samples were inoculated into embryonated chicken eggs. The isolated virus was identified by AGID test and polymerase chain reaction. The virus was isolated from swabs collected from *Grus grus, Balearica pavonina, Numida meleagris*, Duck sp. *Anatidae* and *Pterocles exustus*. Subtyping of the isolated viruses was performed using reverse transcriptase-polymerase chain reaction, which identified the H5 subtype.

**Contribution:**

The present study confirmed the existence and isolation of type A AI virus from different species of wild birds as well as subtyping of its virus for the first time in Khartoum State, Sudan.

## Introduction

Influenza A viruses are grouped in the *Influenza virus A* genus of the Orthomyxoviridae family (Charostad et al. 2023). Avian influenza (AI) viruses are globally prevalent in domestic and non-domestic birds. The viruses are grouped into low-pathogenic (LP) and high-pathogenic (HP) AI viruses by the Office International des Epizooties (OIE 2012). Most of the haemagglutinin–neuraminidase (HA–NA) combinations were identified in avian species, while highly pathogenic avian influenza (HPAI) viruses were detected only in viruses of the subtypes H5 or H7. Highly pathogenic AI virus infection may result in up to 100% losses with drastic production loss in affected domestic poultry and non-domestic birds.

Asymptomatic infections can occur in wild birds; HPAI viruses are seldom detected (Charostad et al. 2023). Waterfowl have been reported to have the highest AI virus prevalence rates, with the greatest subtype variety (Diskin et al. 2020). Wild birds are considered a natural reservoir and may carry LP AI strains. However, viral mutations may lead to the existence of highly pathogenic ones, commonly in H5 and H7 genotypes (Ghersi et al. 2011). All AI type-A subtypes are found in different populations of water birds, which shed large quantities of the virus for long durations in respiratory secretions and/or faeces (Runstadler et al. 2007). The main wild bird species involved in AI are from the orders Anseriformes (geese, swans and ducks) as well as Charadriiformes (especially, waders, gulls and terns) (Olsen et al. 2006).

Despite extensive global knowledge on AI in wild birds, local data from Sudan remain limited. In Sudan, previously a high titre (86.4%) of AI virus type A antibodies was detected in chickens (Ali, Kheir & Ballal 2007), and 28.4% positivity was recorded by competitive enzyme-linked immunosorbent assay (c-ELISA) in Khartoum State (Elamin 2000). Further, sera positive for AI type A were subtyped, 45.3% subtype H9 and 3.5% subtype H7 were detected, while none of the sera tested positive for subtype H5 (Ali & Mansour 2015). Following the highly pathogenic AI virus H5N1 strain outbreak reported in 2006 in Sudan (Ali & Kheir 2007; Ali & Zakia 2015), the detection of AI antibodies in five avian species (domestic fowl, ducks, geese, turkeys and guinea fowls) was reported, with ducks and geese identified as the most affected species (ElNasri & Abdel Rahim 2009). In Sudan, most of the poultry farms are reared in semi-closed and open systems, which facilitate the spread of infectious diseases like AI, either through domestic or wild birds. The previous detection of AI antibodies in wild birds in Sudan suggested its expected role in the transmission of the infection; however, no prior study was done to explore the existence of AI virus antigen and genome in wild birds within Sudan. To aid in the disease control among wild and domestic birds, the present preliminary study objective was to elucidate the existence of type A and subtype H5 AI virus within wild birds in Khartoum State using virus isolation and molecular identification.

## Research methods and design

### Sampling

Six April Zoo is a small zoo with a limited number of animals and birds located within a public park close to the Blue Nile in Khartoum State. During December (the beginning of the winter season), swabs of cloaca and trachea (*n* = 42) have been taken randomly from clinically healthy five different species of wild birds reared in the zoo (Common crane *Grus grus*, Sudan crowned crane *Balearica pavonina*, Helmeted guinea fowl *Numida meleagris*, Duck sp. *Anatidae* and Chestnut-billed sand grouse *Pterocles exustus*). The samples were put in cryotubes containing 1.0 mL phosphate buffer saline (PBS) with antibiotics mixture (penicillin 2000 µ/mL, streptomycin 2 mg/mL, gentamycin 50 µg/mL and 103 IU/mL nystatin). Samples were dispatched to the Virology Department, Central Veterinary Research Laboratory (lab.) (CVRL), Khartoum, stored at −20 °C till being used for virus isolation.

### Detection, isolation and identification of the virus

The collected samples were examined for AI type A virus antigen using agar gel immunodiffusion (AGID) test. Virus isolation was adopted according to the OIE procedure (OIE 2012). Briefly, the prepared inocula were inoculated into the allantoic cavity of 9–10-day-old embryonated chicken eggs and run passages 4–6 times.

### Determination of the isolated virus

#### Serological identification of the isolated virus

**Haemagglutination test:** The existence of haemagglutinating viruses in harvested allantoic fluid was determined using the HA test technique with 1% chicken red blood cell (RBC) suspension. Newcastle disease (ND) virus was excluded by the haemagglutination inhibition (HAI) test against reference ND virus antiserum.

**Agar gel immune diffusion test:** The AI virus identification for the harvested allantoic fluid was examined by AGID test. Allantoic fluids negative for ND virus were tested for avian influenza virus (AIV) with reference AI type A antiserum using AGID test as described (OIE 2012).

#### Molecular identification

To identify the isolated virus, the harvested allantoic fluid was examined for AI type A and subtype H5 using reverse transcriptase-polymerase chain reaction (RT-PCR).

**Reverse transcriptase-polymerase chain reaction:** Reverse transcriptase-polymerase chain reaction, targeting *M* and *HA* genes, was adopted for the identification and subtyping of influenza A virus.

**Ribonucleic acid extraction:** The nucleic acid of the virus collected randomly from the five wild bird species investigated was purified from samples using QIAamp^®^ Viral Ribonucleic Acid (RNA) Mini Kit (Qiagen, Venlo, the Netherlands) as in the manufacturer’s protocol instructions. The extracted RNA was frozen at −80 °C till used.

#### Positive and negative controls

Avian influenza genome of domestic poultry locally isolated strain (Ali & Kheir 2007) was kindly provided by the CVRL, Khartoum. Serial dilutions were used to estimate the optimum deoxyribonucleic acid (DNA) concentration and double-distilled water (DDW) was used as negative control.

#### Synthesis of complementary deoxyribonucleic acid

Synthesis of complementary deoxyribonucleic acid (cDNA) was adopted by transcriptor first-strand cDNA synthesis kit (Roche, Inc., Rotkreuz, Switzerland). Briefly, 1 µL random hexanucleotide primers (600 pmole/µL), 4 µL 5x reaction buffer (8 mM MgCl_2_), 0.5 µL of ribonuclease (RNase) inhibitor (40 U/µL) and reverse transcriptase (20 U/µL), 2 µL 10 mM deoxyribonucleotides (dNTP) mix and 7 µL water free of nuclease were added to 5 µL of deoxyribonucleotides (DNA). The reaction was then completed in accordance with the procedure provided.

#### Amplification of *M* gene

The reaction was performed using primers designed by Capua and Alexander (2009) for AI identification (Table 1). Polymerase chain reaction (PCR) mix includes 5 µL of buffer, 2.5 mM MgCl_2_, 1 mM dNTPs, 100 mM dithiothreitol (DTT), 0.3 µM primers (Table 1), 10 U RNase inhibitor, 2.5 U Taq DNA polymerase (QIAGEN). The PCR condition set was as follows: 42 °C for 20 min, then followed by 95 °C for 5 min; denaturation at 94 °C for 60 s, annealing at 55 °C for 60 s and then 40 cycles of extension at 72 °C for 60 s, with a final elongation at 72 °C for 10 min. Amplicons were detected in ethidium bromide-stained agarose gel (2%).

**TABLE 1:** Sequences of the oligonucleotide primers for detection of and typing of H5 influenza viruses.

Target gene/virus	Primer sequences	Amplicon size (bp)	Reference
*M*/AI	M52:5’-CTT CTA ACC GAG GTC GAA ACG-3’	244	Capua and Alexander (2009)
	M253: 5’-AGGGCATTT TGGACAAAG/T CGTCTA-3’		
*HA*/H5 AI	H5-kha-1: CCT CCA GAR TAT GCM TAYAAA ATT GTC	320	Capua and Alexander (2009)
	H5-kha-3: TAC CAA CCG TCT ACC ATKCCY TG		

Note: Please see the full reference list of the article Ali, W.H., Saeed, I.K., Mutwakil, S.M., Alamin, M.H., Balla, A.A., Ahmed, M.A.E. et al., 2025, ‘Isolation and subtyping of avian influenza A virus from wild birds in Khartoum, Sudan’, *Onderstepoort Journal of Veterinary Research* 92(1), a2228. https://doi.org/10.4102/ojvr.v92i1.2228, for more information

bp, base pair; AI, avian influenza.

#### Haemagglutinin gene amplification

Amplification of the *HA* gene was achieved using primers derived from the published sequences and according to the procedure described by Slomka et al. (2007) (Table 1). The reaction was completed using One-Step enzyme mix (Qiagen One-Step RT-PCR Kit), including primers and 8 U of RNase inhibitor (Promega). The thermal cycle or heating conditions were as follows: for 30 min at 50 °C; 94 °C for 15 min; 40 cycles at 94 °C for 30 s, 58 °C for 1 min and then 68 °C for 2 min and then with a final extension at 68 °C for 7 min. Amplicons were visualised on a 2% agarose gel.

### Ethical considerations

Ethical clearance to conduct this study was obtained from the Central Veterinary Research Laboratory, Department of Virology, Khartoum, Sudan. The material used is only cloacal and tracheal swab specimens collected from clinically healthy wild bird species. The samples collected during the study are only cloacal and tracheal swabs; no administration of any hazardous materials was done to any of the sampled birds.

## Results

### Detection, isolation and identification of the virus

Using AGID, AI type A virus antigen was detected in all tested cloacal and tracheal swabs of wild birds (*Grus grus, Balearica pavonina, Numida meleagris*, Duck sp. *Anatidae* and *Pterocles exustus*). The detected AI virus was isolated from the embryonated chicken eggs.

### Serological identification of the isolated virus

#### Haemagglutination test

The harvested allantoic fluid of the inoculated embryos was found to agglutinate 1% chicken RBCs using HA test.

#### Agar gel immunodiffusion test

Existence of AI virus in the harvested fluids was confirmed by using AGID test.

### Molecular identification of the isolated virus

#### Reverse transcriptase-polymerase chain reaction

**FIGURE 1:**
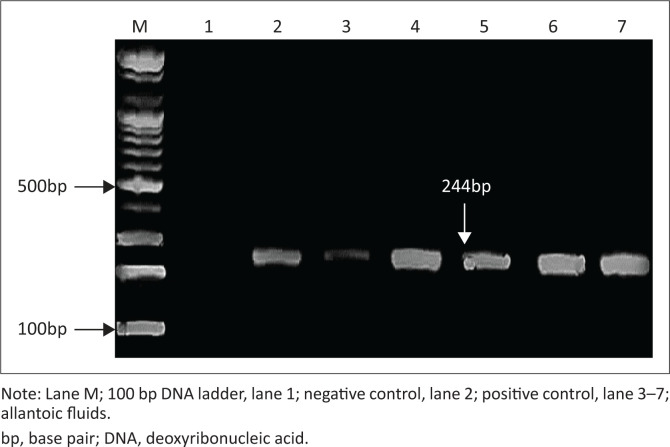
Ethidium bromide-stained agarose gel (2%). Polymerase chain reaction was carried out on ribonucleic acid extracted from allantoic fluids of chick embryos using M52/M253 primers for amplification of avian influenza matrix gene.

**FIGURE 2:**
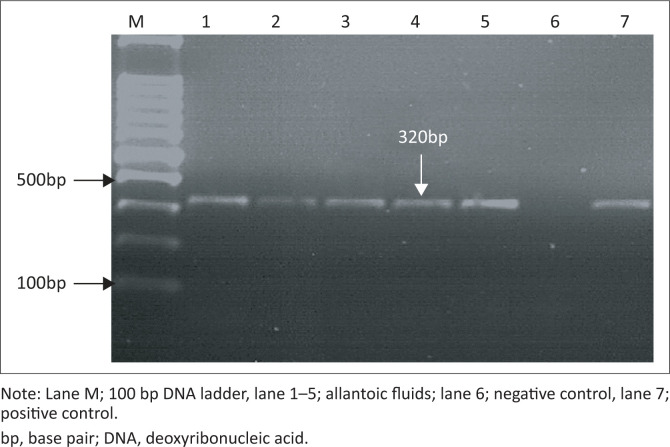
Ethidium bromide-stained gel (2%). Polymerase chain reaction was carried out on ribonucleic acid extracted from allantoic fluids of chick embryos using H5-Kha-1/H5-Kha-3 primers for subtyping of H5 avian influenza.

All samples under test gave positive results. Clear bands were detected in the ethidium bromide-stained gel that correspond to the probable band size for *M* gene (244 base pairs [bp]) and *HA* gene (320 bp). Control positive gave positive results, while no products were amplified when DDW was used as template (Figure 1 and Figure 2).

## Discussion

Wild birds are reported as a natural reservoir for AI virus subtypes (Capua & Alexander 2009; Graziosi et al. 2024; Swayne 2008). Low-pathogenic viruses are the main circulating AI viruses for domestic galliforms with no obvious clinical signs in infected birds. However, these viruses can spread to other birds, where some strains may be adapted (Brown, Poulson & Stallknecht 2014). Mostly, after the establishment of AI virus in a new host, wild birds’ involvement in the viral transmission and maintenance is not significant because of the host adaptation changes, for that, determination of HPAI viruses in wild birds is rare (Brown et al. 2014).

In this study, AI virus type A was detected and isolated in five species of apparently healthy wild birds kept in Six April Zoo at Khartoum State. The AI virus was isolated after two to three blind passages in allantoic cavity of the embryonated chicken eggs. High titres were obtained from passage four up to six, and this might be because of host variation, since the isolated viruses are un-adapted to hen eggs. The isolated viruses were confirmed as AI serologically by AGID test and molecularly by One-Step RT-PCR. The isolated viruses in this study were from five different species of wild birds, including Common crane *Grus grus*, Sudan crowned crane *Balearica pavonina*, Helemeted guinea fowl *Numida meleagris*, Duck sp. *Anatidae* and Chestnut-billed sand grouse *Pterocles exustus*. Our results agreed with many reports, AI naturally occurring infections in over 100 diverse wild birds’ species have been reported. Majority of them are existing in water habitats, particularly species in Anseriformes order (swans, geese, waterfowl and ducks) and Charadriiformes order (shorebirds, terns and gulls) are considered the most important species because of their ecologically documented role as natural reservoirs for influenza virus A (Stallknecht et al. 2012). Birds of different species within these two orders are biologically and behaviourally variable (Pinon & Vialette 2019). This study confirmed the previous reports of AI in wild birds. Most of the reported AI virus isolations from waterfowl were among ducks (Anatinae subfamily), particularly diving and dabbling ducks (Ali & Kheir 2007). In water, AI viruses are relatively stable under certain conditions (Kirunda et al. 2014), suggesting that the aquatic habitat may enhance viral spread between different host species. In Uganda, AI prevalence was the highest in domestic ducks and turkeys, followed by free-living waterfowl (Kirunda et al. 2014). Our results are in line with reports in many countries with various environmental and managemental factors. Avian influenza type A virus detection among ducks was documented in Indonesia (Lestari et al. 2020; Mutisari et al. 2021), across various duck species in different African countries (Kalonda et al. 2020; Kirunda et al. 2014) and in Korea (Lee et al. 2017). Existence of AI type A virus in different species of wild birds was documented, in migratory wild birds in Japan (Abao et al. 2013), ducks in Bangladesh (Khatun et al. 2024; Parvin et al. 2020) as well as wild birds in China (Cui et al. 2020), Germany (King et al. 2021), the Netherlands (Engelsma et al. 2022), Egypt (El-Shesheny et al. 2021; Mahmoud et al. 2024) and the United States (US) (Bevins et al. 2016).

In the present study, subtype H5 was detected for the first time in all subtyped samples collected from the five examined wild bird species. This agrees with reported subtypes in Central, Eastern, Southern and West African countries, where H5 was the most detected subtype (79%) in both domesticated and wild birds (Kalonda et al. 2020; Kirunda et al. 2014). The same results were obtained in wild birds in Korea (Lee et al. 2017) and Japan (Abao et al. 2013). Subtype H5 was found as well in wild birds in China (Cui et al. 2020), Germany (King et al. 2021), the Netherlands (Engelsma et al. 2022), Egypt (El-Shesheny et al. 2021; Mahmoud et al. 2024), the US (Bevins et al. 2016) and in water habitats of birds (Kenmoe et al. 2024).

### Limitation of the study

The study was focused and limited to a single area with a limited number of samples; it was intended only to shed light on the existence of AI infection in wild birds and to open the floor for further epidemiological and molecular studies in the future.

## Conclusion

The present study confirmed the isolation of the virus from five different wild bird species, along with the subtyping of the identified virus. Further routine surveillance and research studies are required to be carried out to determine the virus’s virulence, its molecular characterisation, host susceptibility and the prevalence of viral infection in each species, to aid in the effective control of this viral infectious disease.
